# Student performance of the general physical examination in internal medicine: an observational study

**DOI:** 10.1186/1472-6920-14-73

**Published:** 2014-04-08

**Authors:** Catharina M Haring, Bernadette M Cools, Jos WM van der Meer, Cornelis T Postma

**Affiliations:** 1 Department of Internal Medicine (463), Radboud university medical center, PO Box 9101, 6500 HB Nijmegen, The Netherlands

**Keywords:** General physical examination, Undergraduate medical education, Assessment

## Abstract

**Background:**

Many practicing physicians lack skills in physical examination. It is not known whether physical examination skills already show deficiencies after an early phase of clinical training. At the end of the internal medicine clerkship students are expected to be able to perform a general physical examination in every new patient encounter. In a previous study, the basic physical examination items that should standardly be performed were set by consensus. The aim of the current observational study was to assess whether medical students were able to correctly perform a general physical examination regarding completeness as well as technique at the end of the clerkship internal medicine.

**Methods:**

One hundred students who had just finished their clerkship internal medicine were asked to perform a general physical examination on a standardized patient as they had learned during the clerkship. They were recorded on camera. Frequency of performance of each component of the physical examination was counted. Adequacy of performance was determined as either correct or incorrect or not assessable using a checklist of short descriptions of each physical examination component. A reliability analysis was performed by calculation of the intra class correlation coefficient for total scores of five physical examinations rated by three trained physicians and for their agreement on performance of all items.

**Results:**

Approximately 40% of the agreed standard physical examination items were not performed by the students. Students put the most emphasis on examination of general parameters, heart, lungs and abdomen. Many components of the physical examination were not performed as was taught during precourses. Intra-class correlation was high for total scores of the physical examinations 0.91 (p <0.001) and for agreement on performance of the five physical examinations (0.79-0.92 p <0.001).

**Conclusions:**

In conclusion, performance of the general physical examination was already below expectation at the end of the internal medicine clerkship. Possible causes and suggestions for improvement are discussed.

## Background

The physical examination is a key component of clinical medicine. Critical decisions in patient management often emerge from physical examination findings. Repeated careful physical examination of recently admitted patients has been shown to change diagnoses and treatment in more than one in every four patients [1]. Achievement of proficiency in the performance of a physical examination should therefore be a priority in the training of medical students.

Nevertheless several studies have shown that many practicing doctors are frequently not fully competent in physical examination. For instance, a study on basic physical examination skills of internal medicine residents revealed important deficiencies [2]. Likewise, other studies show deficiencies in interns’ and students’ physical examination skills [3-5]. These studies evoke serious concerns about the general quality of clinical care, since physical examination is a critical factor in clinical decision making.

So, somewhere in the curriculum of the medical student the learning goals regarding physical examination are not met. It is possible that overall decline in physical examination skills already appears in an early stage of medical training, as has been shown for breast examination [6]. In general, students tend to start the clinical clerkships with some level of basic skills, in many curricula acquired during systematic skills training courses in preclinical and in early clinical courses. The internal medicine clerkship is to a large extent the setting in which the actual process of clinical skills acquisition is supposed to take place. As decline in physical examination skills could already occur at this stage, knowledge regarding the level of performance after the internal medicine clerkship is essential.

In our training region we expect students to perform a general screening physical in every encounter with a new patient, regardless of the presenting problem. This instructional design was chosen and implemented as a precaution not to cause cognitive overload in these students. Cognitive overload could easily occur since the students are only at a novice level considering history taking and clinical reasoning at this stage of their training and casus in internal medicine are often complex. The content of this general screening physical examination was previously set by consensus [7]. In this study we analyzed if medical students were able to correctly perform the consensus based core general physical examination regarding completeness as well as technique at the end of the clerkship internal medicine.

## Methods

The physical examinations of 100 medical students were recorded at the completion of their internal medicine clerkship. During the eight weeks of this clerkship they were trained to perform physical examinations in clinical practice with real patients. This clerkship was preceded by systematic clinical skills training of 8 weeks duration. The students were instructed to do a standard general, or core physical examination, as they had learned during the systematic skills training and practiced during the clerkship. The examinations took place in a standard examination room at the outpatient clinic to which they were accustomed. Students had to bring their own instruments, like stethoscopes. Disposable items were provided. They had to examine a specially trained person who was instructed not to express any physical complaints during the examination. No other persons were present in the examination room. A camera was positioned at the head end of the examination bed.

In a previous study we reported the consensus the trainers in our region had reached on the components of the physical examination that should be included in a standard general or core physical examination [7]. At the time of the study, this consensus was constructed but not yet integrated in physical examination training. Therefore we could then analyze whether students perform a physical examination as generally expected at that time. An adapted checklist containing 59 items was constructed. The adapted checklist contained four more items than the original list. Two original items (reflexes of the upper extremities and reflexes of the lower extremities) were subdivided to be able to observe performance of the individual maneuvers. One last item (measuring blood pressure on both arms) was added after group discussion with regional trainers. A list of 30 extra items not included in the standard was added to the checklist to facilitate the recording of extra performed items. For each standardized item a short description was included as to how it should be performed in accordance with the preferred textbook and linked website with examples of the physical examination on each topic [8]. These descriptions were considered the gold standard and were used for rater instruction. In the checklist the performance of the physical examination items could be scored as: adequate, inadequate or technique not completely visible and assessable. Inspection was only taken into account as indeed performed if students clearly spent time to inspect a certain part of the body. One physician (CH) noted the frequency and adequacy of performance of all components of the physical examination for 100 students. For analysis of the reliability of the observations, five physical examinations were randomly selected and rated by three physicians, all experienced in physical examination training. Inter-rater reliability was estimated by calculation of the intra-class correlation coefficient for total scores of the physical examinations and for agreement on performance of all 59 items per physical examination. Intra-class correlation coefficients were computed, using an absolute agreement definition. All statistics were calculated using the SPSS 16.0 package program.

Total scores of the five physical examinations were computed as follows: Students received two points for an adequately performed item, one point for an inadequately performed item and no points when an item was omitted. When an item was done but the technique was not assessable on the recording, the item was registered as missed value and not included in the analysis.

Student participation was on a voluntary basis. The standardized patients were all actors that regularly participate in physical examination training sessions. They were all asked informed consent before participation. Approval of the institutional ethical review board was not required according to the guidelines of the institutional research board based on the Dutch law (WMO).

## Results

The 100 recordings were assessed and analyzed. In general the students performed a physical examination that was less extensive than expected by their supervisors (Table 1). On average 36 of the 59 items were completed. The frequency with which an item was performed varied widely between 100 and 0 times. The percentage of students conducting an item adequately varied considerably between the items. There were eight items, listed in Table 2, not included in the standard physical examination that were done extra by more than five percent of the students.

**Table 1:** Actual student performance of the general physical examination in internal medicine

**Maneuver**	**Performed n = 100**	**Performed adequate (%), if technique assessable**	**Performed, technique not assessable n = 100**
Auscultation of the abdomen	100	82	0
Measuring blood pressure	100	77	0
Percussion of the lungfields	100	57	5
Palpation of the lymph nodes of the head and neck	100	54	15
Inspection of the oral cavity and pharynx	100	9	6
Auscultation of the lungfields	99	77	2
Light and deep palpation of the abdomen	99	57	0
Auscultation of the heart	98	91	0
Percussion of the abdomen	98	61	0
Palpation of the dorsalis pedis artery	97	100	1
Palpation of the liver	97	41	0
Assessment of the level of diaphragmatic dullness	95	82	10
Counting pulse rate	94	97	0
Palpation of the posterior tibial artery	92	95	2
Percussion of the liver span	91	80	0
Palpation of the spleen	91	45	2
Patellar tendon reflex	91	94	3
Testing pupillary light reflex	91	92	9
Assessment of sinus tenderness	87	99	6
Auscultation of the carotid artery	85	99	2
Palpation of the femoral artery	85	82	0
Assessment of extraocular muscle strength	85	68	4
Achilles tendon reflex	83	84	16
Biceps tendon reflex	80	90	0
Measuring jugular venous pressure	78	46	8
Measuring weight	76	100	0
Palpation carotid artery	75	99	3
Examination of the thyroid gland	75	75	3
Plantar reflex	73	99	1
Measuring height	72	100	0
Inspection of the conjunctiva	72	100	3
Percussion of the heart	71	37	0
Percussion of the spleen	68	40	0
Measuring blood pressure standing	65	62	2
Assessment of cardiac movements	64	98	0
Measuring blood pressure on both arms	61	98	0
Auscultation of the femoral artery	56	91	0
Palpation of the popliteal artery	56	77	0
Examination of peripheral edema	48	79	0
Assessment of kidney tenderness	43	100	0
Palpation of the inguinal lymph nodes	38	71	0
Percussion of the spine	37	100	0
Palpation of the axillary lymph nodes	36	63	4
Global inspection of the extremities	31	39	0
Palpation of the abdominal aorta	30	43	0
Inspection of the abdomen	22	95	0
Assessment of skin turgor	19	90	0
Inspection of the chest	16	94	0
Triceps tendon reflex	11	63	0
Palpation of the breasts	9	56	0
Counting breathing frequency	6	100	0
Percussion of the bladder	5	100	0
Applying vertical pressure to the spine	2	100	0
Total inspection of the skin	2	50	0
Assessment of sensibility	2	0	0
Inspection of the breasts	1	100	0
Assessment of the curvature and mobility of the spine	1	0	0
Assessment of strength of the upper extremities	1	0	0
Assessment of strength of the lower extremities	0	0	0

**Table 2:** Extra performed items

**Extra performed physical examination item**	**Percentage of students**
Assessment of nasal patency	81
Testing the near reflex	39
Palpation of radial artery on both sides	23
Palpation of brachial artery on both sides	21
Inspection and palpation of the scalp	8
Inspection of the ears	8
Palpation of the calves	7
Palpation of the kidneys	7

Physical examination procedures not included in the standard physical examination, performed by more than 5% of the students.

The performance of the items was in the majority of cases easily visible on the recording (Table 1). Only 107 (2.9%) of the items could not be adequately assessed, mostly caused by the students positioned between the patient and the camera. Inter-rater reliability expressed as the intra-class correlation coefficient for the total scores of the physical examinations was 0.91 (p <0.001). For five physical examinations rated by three different physicians this ranged from 0.79 to 0.92 (p < 0.001) per physical examination scored (Table 3).

**Table 3:** Interrater reliability

**Performed physical examination**	**ICC**	
Student 1	0.87	P < 0.001
Student 2	0.79	P < 0.001
Student 3	0.85	P < 0.001
Student 4	0.82	P < 0.001
Student 5	0.92	P < 0.001

Interrater reliability for five complete physical examinations of five randomly chosen students rated by three physicians.

## Discussion

This study shows that medical students in general did not perform an adequate standard physical examination at the end of the internal medicine clerkship. Approximately 40% of the items that should be performed were omitted, while many items were performed incorrectly. This is a relevant and important problem. The standard by which the students were assessed was a core physical examination defined by the clinical teachers and practicing physicians of our university medical centre and its affiliated clinics. A full head-to-toe exam is at present under reconsideration in many respects, therefore a 55-item core exam covering the most important topics as basis for a further analysis and management plan could be seen as a reasonable to fulfill goal during the initial clinical training. In this study the students were instructed to perform a physical examination of a simulated patient as they themselves deemed adequate. As far as we know this is the first description of such a set-up at the end of a clerkship.

The outcome of this study should raise concern. During the clerkship of internal medicine, students are supposed to practice and ultimately master the general physical examination. It appears that deficiencies in the execution of the physical examination are nevertheless already present at the end of the basic training in internal medicine. What is worrisome is that some elements defined as components of a core physical examination were omitted by over 90% of the students. The students involved in this research knew they were being observed, and it is therefore very possible that they performed an even more thorough examination than otherwise would have been done under routine circumstances. It is known that physicians perform a more extensive physical examination in an examination setting as opposed to what they actually do when they are observed in a doctor-patient encounter [9].

Inter-rater reliability with respect to total scores of the physical examinations was excellent. Indicating that producing a total score of a complete physical examination by this checklist, consisting of many items, could be done reliably. This has been shown before in a larger study where an inter-rater variability of total encounter scores of an 138-item head-to-toe physical examination by trained patient instructors showed an intra-class correlation coefficient of 0.95 [10]. We were also able to reach high intra-class correlation coefficients for absolute agreement on performance of individual total physical examinations. This implies that, when using experienced raters, performance of an individual total physical examination can be reliably assessed as a whole and provides solid ground for feedback.

There appear to be various underlying potential causes for the discrepancy between expected and actual performance in physical examination in this early phase of clinical training. From a previous study we know that students will be confronted with different opinions of their supervisors concerning the extent of the physical examination that should be performed when examining a patient for the first time. In addition, students may see their supervisors perform less extensive physical examinations [7]. What adds to the problem is that the student’s interaction with patients is rarely observed. Deficiencies arising during the clerkships will not be noted and corrected and hence there is possibly little direct formative feedback during and about patient contacts. We know that in practice direct observation of physical examination skills appears not to be standard practice [11,12]. Some researchers even noted that 80% of students reported never being observed by faculty while performing a complete physical examination [13]. Apparently, supervising physicians tend to rely mainly on the written or oral report of the physical examination presented by the student and assume it was complete and accurately performed [14].

### Limitations

In our university medical centre the clerkship in internal medicine is preceded by two four week courses of systematic skills training including communication and history taking, clinical reasoning and physical examination. Students must pass a 12 station objective structured clinical examination (OSCE) in order to be admitted to the next part of the clerkships which is an introductory clerkship. Physical examination is an integral part of this OSCE. Thereafter the students follow a 4 week introductory course in a clinical setting during which they are intensively observed in their patient encounters and also assessed in their clinical competence including physical examination. The training in these courses is provided by experienced clinical teachers. Only if they also pass this training successfully are they admitted to the internal medicine clerkship. Although we presuppose that competence in physical examination at the beginning of the clerkship is accurate given all of this training, this was not determined by the same extended test procedure as we used in this study.

The study did not take place in real time by observation of real patient encounters during the clinical clerkship. For organizational reasons, privacy and ethical concerns we chose for a set-up with standardized patients. This may have influenced the behavior of the students. However, to limit this effect, the setting was the clinical environment to which the students were accustomed.

Students were not aware of the test criteria when they participated in the study. It could be argued that they may have performed better if they had been informed. Our argument for the taken strategy was to get a better reflection of what they actually do in practice. We argued that if we provided the criteria, they would act upon these and not perform as if in real practice.

Inspection of certain body parts was part of the core physical examination. Inspection was only taken into account when a student clearly took time to inspect the body part. It is possible that some inspections were missed by the researchers. It is hard to determine whether this has happened in our study. Asking students afterwards which body parts had been inspected, could have generated socially accepted answers. It is more likely that inspection was not consciously done in most cases.

### Recommendations

What can be done to improve physical examination skills? First of all, students and teachers should be made aware of what is expected regarding student performance during the clerkships. Uniformity could be enhanced by distribution of (electronic) pocket cards containing the standard for physical examination. Diverging opinions of teachers lead to great confusion and should be avoided [15]. In addition, awareness should be raised among faculty that omissions in the performance of a physical examination by medical students can only be uncovered by actual observation of the student. Observation of the physical examination should be obligatory and not an option. Finally, a skills improvement program throughout the clinical clerkships might increase the frequency in which physical examination is used, as has been shown previously for residents [16]. If students cannot perform this core exam completely or correctly, they may not develop the necessary range of normal findings by own experience, and may be developing incorrect habits that will remain with them in their clinical practice. Even residents are only rarely directly observed. This indicates a need for continuous monitoring of the development of proficiency of the physical examination skills and remedial teaching if necessary.

## Conclusion

Performance of the general physical examination was already below expectation at the end of the internal medicine clerkship.

## Competing interest

The authors declare that they have no competing interest.

## Authors’ contributions

CH participated in de design of the study, acquisition of data, analyzed the data, and drafted the manuscript. JM participated in the design of the study and revised the manuscript. BC participated in acquisition and interpretation of data. CP participated in the design of the study, acquisition of data, coordination of the study, and revised the manuscript. All authors have read and approved the manuscript.

## Authors’ information

Catharina M Haring, MD is a nephrologist and working as a Researcher in Medical Education at the Radboud university medical center. Her main research interest is assessment of clinical competence in Internal Medicine.

Cornelis T Postma, MD PhD is associate professor of Medicine and principal lecturer in medical education at the department of Internal Medicine of the Radboud university medical center. His main medical education interests are in the training of practical medical education and medical competence, and in the field of clinical assessment.

Jos WM van der Meer, MD, PhD is emeritus Professor of Medicine, principle lecturer in medical education and former head of the department of Internal Medicine of the Radboud university medical center.

Bernadette M Cools, MD is an internist and junior principal lecturer in medical education at the department of Internal Medicine of the Radboud university medical center. Her main medical education interest is preclinical systematic skills training.

## Pre-publication history

The pre-publication history for this paper can be accessed here:

http://www.biomedcentral.com/1472-6920/14/73/prepub
